# Identification of markers for predicting prognosis and endocrine metabolism in nasopharyngeal carcinoma by miRNA–mRNA network mining and machine learning

**DOI:** 10.3389/fendo.2023.1174911

**Published:** 2023-07-19

**Authors:** Xixia Zhang, Xiao Li, Caixia Wang, Shuang Wang, Yuan Zhuang, Bing Liu, Xin Lian

**Affiliations:** ^1^ Department of Otolaryngology Head and Neck Surgery, Shengjing Hospital of China Medical University, Shenyang, China; ^2^ Department Obstetrics and Gynecology, Shengjing Hospital of China Medical University, Shenyang, China

**Keywords:** nasopharyngeal cancer, micro RNAs, miRNA-mRNA network, immunotherapy, immune checkpoint blockade, risk model, endocrine

## Abstract

**Background:**

Nasopharyngeal cancer (NPC) has a high incidence in Southern China and Asia, and its survival is extremely poor in advanced patients. MiRNAs play critical roles in regulating gene expression and serve as therapeutic targets in cancer. This study sought to disclose key miRNAs and target genes responsible for NPC prognosis and endocrine metabolism.

**Materials and methods:**

Three datasets (GSE32960, GSE70970, and GSE102349) of NPC samples came from Gene Expression Omnibus (GEO). Limma and WGCNA were applied to identify key prognostic miRNAs. There were 12 types of miRNA tools implemented to study potential target genes (mRNAs) of miRNAs. Univariate Cox regression and stepAIC were introduced to construct risk models. Pearson analysis was conducted to analyze the correlation between endocrine metabolism and RiskScore. Single-sample gene set enrichment analysis (ssGSEA), MCP-counter, and ESTIMATE were performed for immune analysis. The response to immunotherapy was predicted by TIDE and SubMap analyses.

**Results:**

Two key miRNAs (miR-142-3p and miR-93) were closely involved in NPC prognosis. The expression of the two miRNAs was dysregulated in NPC cell lines. A total of 125 potential target genes of the key miRNAs were screened, and they were enriched in autophagy and mitophagy pathways. Five target genes (E2F1, KCNJ8, SUCO, HECTD1, and KIF23) were identified to construct a prognostic model, which was used to divide patients into high group and low group. RiskScore was negatively correlated with most endocrine-related genes and pathways. The low-risk group manifested higher immune infiltration, anticancer response, more activated immune-related pathways, and higher response to immunotherapy than the high-risk group.

**Conclusions:**

This study revealed two key miRNAs that were highly contributable to NPC prognosis. We delineated the specific links between key miRNAs and prognostic mRNAs with miRNA–mRNA networks. The effectiveness of the five-gene model in predicting NPC prognosis as well as endocrine metabolism provided a guidance for personalized immunotherapy in NPC patients.

## Introduction

Nasopharyngeal cancer (NPC) is an epithelial malignancy, which has discrepant occurrence in different regions and countries. The etiology of NPC is multiple and remains incompletely understood, but most cases are closely linked to Epstein–Barr virus (EBV) infection (1). In Western countries, the incidence rate is relatively low with a ratio of 1:100,000 each year. However, in the regions of Southern China and Asia, the annual incidence elevates to 25–50 cases per 100,000 (2), which accounts for approximately 70% new cases worldwide (3). The incidence also strikingly increases in the recent decades in China. The age-standardized incidence rate (ASIR) was 3.3/100,000 in 1990, whereas it reached 5.7/100,000 in 2019. The rising incidence rate was especially startling in men, with ASIR of 4.3/100,000 to 8.6/100,000 from 1990 to 2019 (4). The distant metastasis contributes to an extremely poor prognosis in NPC patients, and the patients of stages III and IV have a 5-year survival rate less than 10%.

With intensive usage of modulated treatment including chemotherapy and radiotherapy, the control of NPC metastasis reaches a satisfactory outcome and the 5-year survival rate drastically improves (5–7). Nevertheless, the prognosis and treatment efficiency were awfully unfavorable in the NPC patients of late stages. It is still a great challenge for clinicians to control and lessen the metastasis and recurrence of advanced NPC patients. In the recent years, immunotherapy reaches a milestone in clinical cancer therapy, not excluding in NPC. Immune checkpoint blockade (ICB) therapy is one of the promising strategies to increase antitumor activity in NPC. Studies have shown that NPC patients expresses high expression levels of programmed death protein 1 (PD-1) and programmed death ligand 1 (PD-L1) that are associated with poor outcomes and recurrence (8, 9). The blockade of PD-1/PD-L1 expression can recover the ability of cytotoxic lymphocytes exerting anticancer response. Lines of clinical trials of ICB therapy have demonstrated the positive response to anti-PD-1/PD-L1 drugs such as pembrolizumab in phase 1 and 2 studies (10, 11). In the phase 2 study, of 44 enrolled NPC patients, eight patients reached a partial response and one patient reached a complete response, showing an objective response rate (ORR) of 20.5% (11). As we know, in the tumor microenvironment, the expression of PD-1 on the surface of tumor cells and the combination of PD-L1 on the surface of tumor-infiltrating lymphocytes can inhibit the activity of T cells, lead to the loss of function of effector factors TNF-a, IFN-gamma, and IL-2, and inhibit cytolysis function through granulozyme B and perforin, and ultimately promote immune escape (12, 13). Obviously, still a high proportion of NPC patients showed a negative response to ICB therapy, which may result from their disadvantageous tumor microenvironment. Therefore, in order to raise the treatment accuracy, understanding the mechanism of immune evasion and developing molecular biomarkers is critically needed.

Over the last few decades, it has become clear that endocrines are also involved in regulating tumor cells and that cancer cells themselves abnormally express and respond to many hormones (14). Both leptin and its receptor have been reported in cancer biopsy specimens, indicating autocrine and/or paracrine roles in tumorigenesis (15). Several hypothalamic hormones have been implicated in a variety of human cancers, including growth hormone-releasing hormone, luteinizing hormone-releasing hormone, somatostatin, and bombotin (16, 17). It is becoming increasingly clear that many human cancer cells are sensitive to a variety of hormones and that they themselves express many hormones that play an important role in the development and progression of cancer.

Non-coding RNAs play essential roles in gene modulation and pathway regulation. Some microRNAs (miRNAs) were unveiled to participate in NPC invasion and metastasis, immune escape, and resistance to chemotherapy and radiotherapy (18), endowing miRNAs possible to serve as potential therapeutic targets (19). For example, miR-26a was found to have an anticancer effect and overexpressing miR-26a could inhibit the metastatic feature in NPC cells (20). MiR-663 targeting WAF1/CIP1 promotes the proliferation and tumorigenesis in NPC cells (21). The crosstalk between extracellular microRNAs and the tumor microenvironment has also been profoundly parsed by previous studies (22, 23). Consequently, our study tried to mine the key miRNAs that had a prognostic value in NPC. We identified two potentially key miRNAs (has-miR-142-3p and has-miR-93) closely involved in NPC prognosis. By building competing endogenous RNA (ceRNA) networks using multiple miRNA tools, we determined 125 potential target genes (mRNAs) and screened five key genes contributable for the prognostic model. The five-gene prognostic model manifested a satisfactory performance in prognosis prediction and estimating the response to immunotherapy and chemotherapy.

## Materials and methods

### Data source and preprocessing

GSE32960, GSE70970, and GSE102349 datasets containing the expression profiles of NPC samples were accessed from the Gene Expression Omnibus (GEO) database (24), where GSE32960 and GSE70970 include miRNA expression data and GSE102349 includes mRNA expression data. We screened the samples of GEO datasets according to the following criteria: 1) remove samples without survival time and survival status; 2) convert probes into gene symbols; 3) remove a probe matching to multiple genes; 4) select the averaged expression of a gene with multiple probes; 5) for miRNA data, only human samples were remained. After preprocessing, 312 NPC and 18 normal samples were remained in the GSE32960 dataset; 253 NPC and 10 normal samples were remained in the GSE70970 dataset; and 88 NPC samples were remained in the GSE102349 dataset.

### Identification of NPC-associated differentially expressed miRNAs

In the GSE32960 dataset, differentially expressed miRNAs (DEmiRNAs) were screened by Limma R package (25) from NPC and normal samples under conditions of P < 0.05 and |log2(fold change, FC)| > 1.2. Gene modules were identified by weighted correlation network analysis (WGCNA) (26). Firstly, samples were clustered and the co-expression network was constructed. A scale-free network was ensured under scale-free R2 = 0.85, and soft threshold (power) = 3 was determined. The co-expression network was then converted to the adjacent matrix and was further converted to the topological overlap matrix (TOM). Subsequently, we clustered genes by the dynamic cutting method and average-linkage hierarchical clustering based on TOM. Eigengenes were calculated for each gene, and gene modules were clustered and merged under parameters of deepSplit = 2, and minModuleSize = 60, height = 0.25. The module–trait relationships were assessed by Pearson correlation analysis. By overlapping the miRNAs in the NPC-associated gene modules and DEmiRNAs, NPC-associated miRNAs were determined.

### Construction of a miRNA-based risk model

Random grouping of samples from the GSE32960 dataset into training group and test group at a ratio of 3:2 was performed. Two-group differences were assessed using Student’s t test. Univariate Cox regression analysis screened NPC-associated miRNAs from the training group. MiRNAs with P < 0.01 were selected as prognostic miRNAs. Multivariate analysis (stepAIC) was introduced to measure the coefficients of prognostic miRNAs. Then, the miRNA-based risk model was defined as: risk score = Σ(coef i*expression i), where coef indicates the coefficients of miRNAs and i represents miRNAs.

### Evaluation and optimization of the risk model

Each sample obtained a risk score calculated by the risk model. The median risk score was employed in dividing samples into low-risk and high-risk groups. Receiver operating characteristic (ROC) curve analysis was used to predict the efficiency of the risk model in predicting overall survival through timeROC R package (27). The prognosis difference of two risk groups was studied by Kaplan–Meier survival analysis. Univariate and multivariate Cox regression models were used to analyze the hazard ratio of risk type. A nomogram was used to optimize the clinical use of the risk model with the rms package.

### Construction of a mRNA-based prognostic model

First of all, the potential target genes of miRNAs were predicted by different online tools and software including microT (28), miRanda (29), mircode (30), miRDB (31), miRmap (32), miRtarbase (33), PicTar (34), PITA (35), TargetMiner (36), TargetScan (37), RNA22 (38), and starbase (39). The target genes predicted by at least six tools were remained as key potential target genes. The WebGestaltR package (40) was utilized to annotate the enriched Kyoto Encyclopedia of Genes and Genomes (KEGG) pathways and Gene Ontology (GO) terms. Then, we used the key target genes to establish the mRNA-based prognostic model. Prognostic target genes in 70% samples of the GSE102349 dataset were screened by univariate Cox regression analysis. Random sampling with 70% samples in the GSE102349 dataset was performed for 1,000 times corresponding with univariate analysis. The top five frequent target genes from the results of 1,000 times of univariate analysis were selected as the final target genes for constructing the mRNA-based prognostic model (defined by the same formula with the miRNA risk model).

### Endocrine metabolism analysis

There were 31 SECRETORY_PATHWAYS screened from the KEGG PATHWAY Database (https://www.genome.jp/kegg/pathway.html). SECRETORY_PATHWAYS scores in the GSE102349 dataset were calculated by ssGSEA (41). There were 26 SECRETORY-related genes obtained from KEGG (https://www.genome.jp/dbget-bin/www_bfind_sub?mode=bfind&max_hit=1000&locale=en&serv=kegg&dbkey=genes&keywords=Secretory&page=1). Pearson analysis was conducted to determine the correlation between SECRETORY_PATHWAYS scores/SECRETORY-related genes and RiskScore.

### Analysis of immune characteristics

We used ssGSEA (41) to estimate the immune cell proportion in two risk groups. The gene sets of 22 immune-related cells, innate immune response, and adaptive immune response were obtained from Charoentong et al. (42). The ESTIMATE algorithm (43) was employed to measure immune cell infiltration and stromal cell infiltration. The ESTIMATE score represents the combined score of immune score and stromal score. MCP-counter (44) was used to evaluate 10 immune-related cells including monocytic lineage, CD3+ T cells, NK cells, CD8+ T cells, endothelial cells, cytotoxic lymphocytes, B lymphocytes, neutrophils, fibroblasts, and myeloid dendritic cells based on the expression matrix. The immune checkpoint genes were downloaded from a previous study (42).

### Assessment of biological pathways

Biological pathways were accessed from “h.all.v7.4.symbols.gmt” downloaded from the Molecular Signatures Database (MSigDB) (45). There were 13 tumor-related genes obtained from a previous research (46), which are classic cancer pathways, involved in the development and progression of cancer, including DNA damage repair (DDR), epithelial–mesenchymal transition (EMT), cell cycle, mismatch repair, CD8 T effector, FGFR3, nucleotide excision repair, base excision repair, DNA replication, homologous recombination, and WNT target. ssGSEA was performed to determine the enrichment score of biological pathways. The relation of risk score with pathways was inspected with Pearson correlation analysis.

### Predicting the response to immunotherapy and chemotherapy

We conducted SubMap analysis (47) to compare the expression profiles between GSE102349 and IMvigor210. IMvigor210 contains the expression profiles of patients with metastatic urothelial carcinoma treated by PD-L1 inhibitors (48). The higher similarity of samples in GSE102349 with complete response (CR) and partial response (PR) groups in IMvigor210 suggested that the samples were more sensitive to anti-PD-L1 treatment. The TIDE (http://tide.dfci.harvard.edu/) algorithm (49) was employed to predict the escape and response to immune checkpoint inhibitors, according to the score of T-cell dysfunction and exclusion, the enrichment of immunosuppressive cells. The sensitivity of two risk groups to chemotherapeutic drugs was estimated using the pRRophetic R package (50).

The performance of the prognostic model was further examined in immunotherapy datasets including IMvigor210, GSE135222, and GSE78220. GSE135222 contains non-small cell lung cancer patients treated with immune checkpoint inhibitors (51). GSE78220 includes patients suffering from metastatic melanoma treated by anti-programmed cell death protein 1 (anti-PD-1) therapy.

### Cell culture

After resuscitating NPC cells and normal cells, they were placed in 1,640 and 5A cell medium containing 10% fetal bovine serum, respectively, at 37°C, 5% CO_2_ concentration, and constant temperature to around 80%–90%, then passed and spread on a plate.

### RT-qPCR

Total RNA was extracted from cells by RT-qPCR, and cDNA was synthesized by reverse transcription. After reverse transcription, samples were added according to the experimental instructions. The reaction conditions of RT-qPCR were 95°C for 30 s. 95°C, 5 s; 60°C, 30 s, 40 cycles. The mRNA expressions of miR-142-3p and miR-93 in the experimental group and control group were analyzed.

### Statistical analysis

The statistical methods used in this study were performed in R software (v4.2.0). The Sangerbox platform (52) was used to provide an assistant in data analysis. The log-rank test was applied in survival analysis. Difference between two groups was examined by the Wilcoxon test. The Kruskal–Walls test was used to test the difference among over two groups. P < 0.05 was determined as statistically significant.

## Results

### Identification of DEmiRNAs related to NPC based on WGCNA

**Figure 1:**
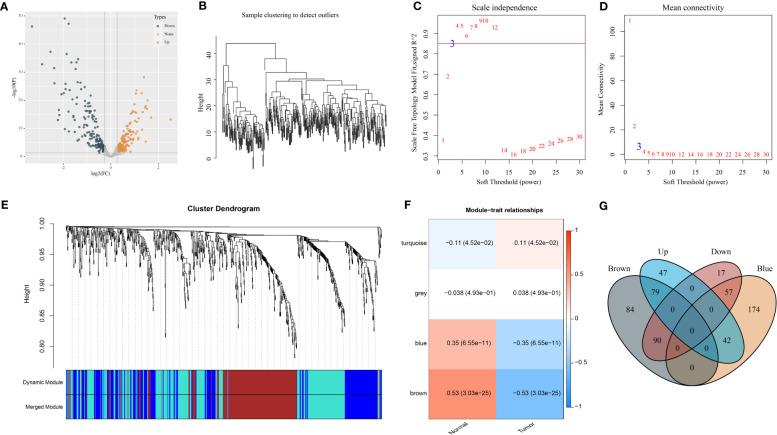
Identification of NPC-related DEmiRNAs in the GSE32960 dataset. **(A)** Volcano plot of 332 DEmiRNAs. **(B)** Clustering dendrogram of 312 samples. **(C, D)** Under different soft thresholds (power), we analyzed scale independence and mean connectivity. **(E)** Clustering dendrogram of DEmiRNAs based on TOM and dynamic cut methodology. **(F)** The relationships of modules with normal and tumor groups. **(G)** Venn plot of DEmiRNAs and miRNAs of brown and blue modules.

To begin with, we assessed the expression difference of miRNAs between normal and tumor samples in the GSE32960 dataset. The results showed that 332 miRNAs were differentially expressed between normal and tumor groups, including 168 upregulated and 164 downregulated miRNAs in tumor groups (**Figure 1A**; **Table S1**). Then, we applied WGCNA to cluster samples and dig out key gene modules based on DEmiRNAs. To ensure the co-expression network to be a scale-free network, the Pearson coefficient was selected as 0.85 and the power of the soft threshold was determined as 3 to construct an adjacency matrix (**Figures 1B–D**). Next, a topology overlap matrix (TOM) was generated based on the adjacency matrix and the genes were divided into different modules using the Dynamic Tree Cut algorithm (**Figure 1E**). Four modules were finally determined after merging the adjacent modules. Then, we analyzed the correlation of four modules with different sample groups. As a result, brown and blue modules were found to be evidently associated with sample groups and they manifested opposite correlations with two groups (**Figure 1F**). Specifically, blue and brown modules were negatively correlated with the tumor group (R = -0.35 and -0.53, respectively). The Venn plot was constructed to describe the intersection between DEmiRNAs and miRNAs of blue and brown modules (**Figure 1G**). There were 99 DEmiRNAs including 42 upregulated and 55 downregulated found in the blue module. There were 169 DEmiRNAs including 79 upregulated and 90 downregulated found in the brown module. The above total 268 DEmiRNAs were determined as potential miRNAs associated with NPC.

### Establishment and verification of a miRNA-related risk model

We randomly divided 312 NPC samples of the GSE32960 dataset at a ratio of 3:2 into the training and test groups (**Table S2**). We used the training group to screen prognostic miRNAs from 268 DEmiRNAs according to the univariate Cox regression model. The analysis identified two miRNAs (hsa-miR-142-3p and hsa-miR-93) that were significantly associated with the overall survival (P < 0.01). Based on the multivariate coefficients of two miRNAs by stepAIC analysis, we established the risk model defined as follows (**Figure S1A**).

Risk score = -0.835*(hsa-miR-142-3p) + 0.85*(hsa-miR-93)

Moreover, the RT-qPCR analysis results showed that miR-142-3p expression was downregulated and miR-93 was upregulated in five NPC cell lines in comparison with normal NP69 cells (**Figures S1B, C**).

**Figure 2:**
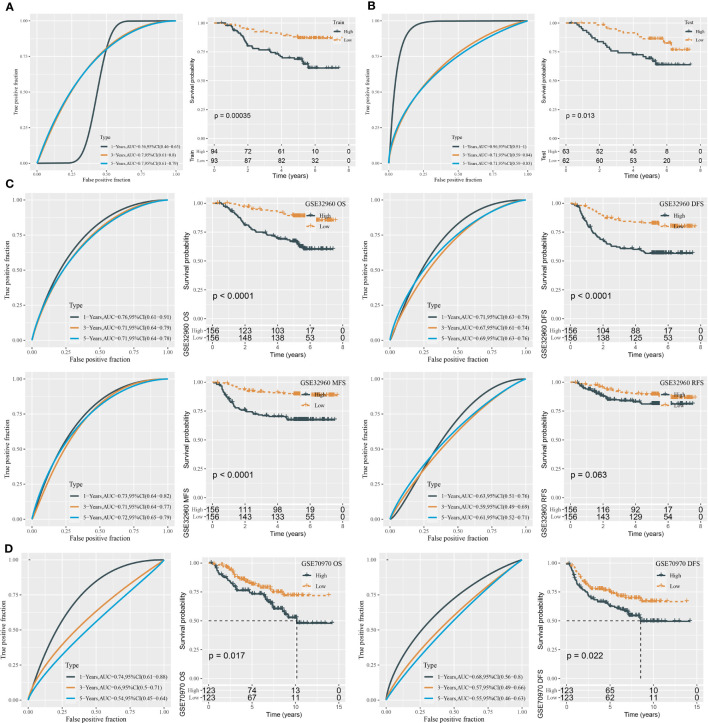
ROC analysis and survival analysis for evaluating the performance of the miRNA risk model. **(A)** ROC curves and survival curves in the training group. **(B)** ROC curves and survival curves in the test group. **(C)** ROC curves and survival curves of DFS, OS, MFS, and RFS in the GSE32960 dataset. **(D)** ROC curves and survival curves of OS and DFS in the GSE70970 dataset.

To validate the performance of the risk model, we calculated the risk score for each tumor sample in the training and test groups. The AUC for 1-, 3-, and 5-year survival derived from the ROC curve analysis was 0.56, 0.70, and 0.70 in the training group and 0.96, 0.71, and 0.71 in the test group, respectively (**Figures 2A, B**). Furthermore, based on the median risk score, tumor samples were classified into low-risk and high-risk groups. The two risk groups had distinct overall survival (OS) in the training and test groups, as shown by Kaplan–Meier survival analysis (P = 0.00035 and P = 0.013, respectively, **Figures 2A, B**).

**Figure 3:**
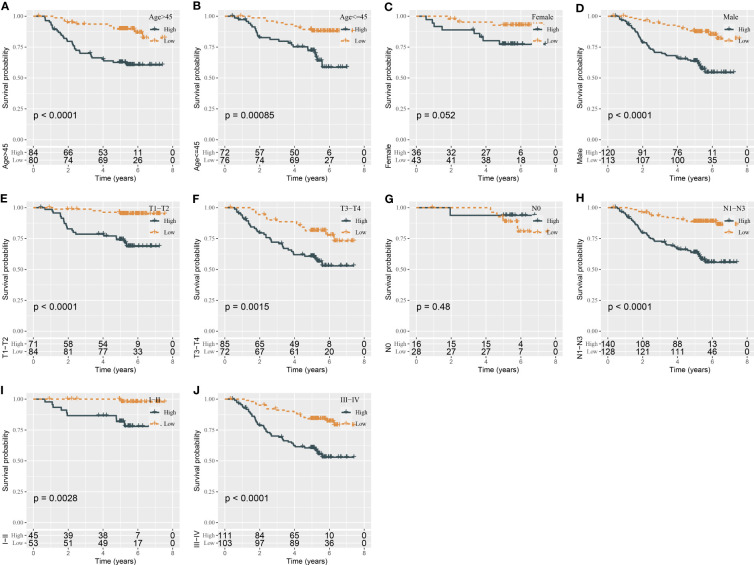
Kaplan–Meier survival analysis of two risk groups in the samples with different clinical characteristics **(A–J)**.

In the total GSE32960 dataset, the risk model still showed good performance in predicting overall survival (**Figure 2C**). In addition, we evaluated the effectiveness of the risk model in different survival times including recurrence-free survival (RFS), disease-free survival (DFS), and metastasis-free survival (MFS). High-risk and low-risk groups exhibited differential prognosis of DFS and MFS (P < 0.0001) but showed no significant difference in RFS classification (P = 0.063, **Figure 2C**). In another independent dataset (GSE70970), the risk model also showed an effect classification for both OS and DFS (P = 0.017 and P = 0.022, respectively) (**Figure 2D**). Moreover, we verified the effectiveness of the risk model in the groups of different clinical characteristics. In addition to the female group and N0 group, the risk model was sufficient to distinguish samples into different risk levels in the groups of age >45, age ≤45, male, T1–T2, T3–T4, N1–N3, I–II, and III–IV (**Figure 3**).

### Boosting the prediction efficiency of the miRNA-related risk model by constructing a nomogram

**Figure 4:**
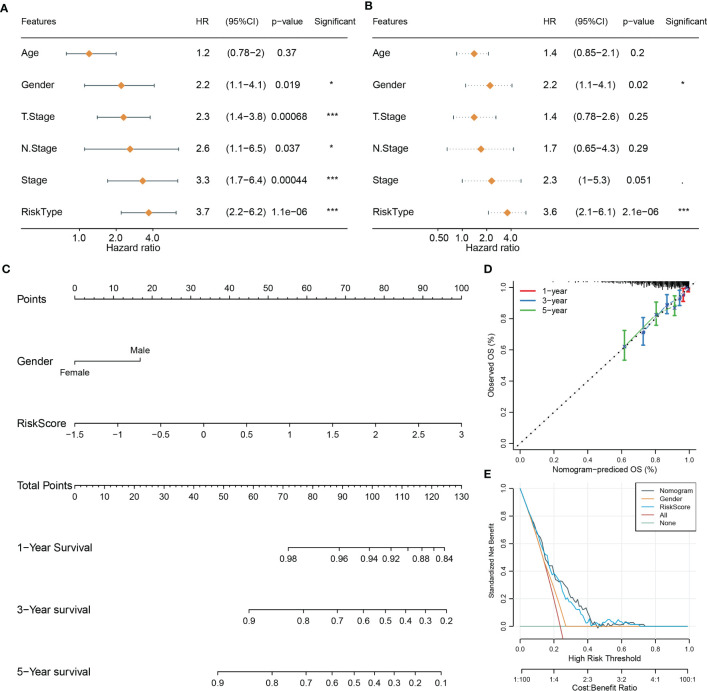
Constructing a nomogram based on clinical features and risk score. **(A, B)** Univariate **(A)** and multivariate Cox regression analyses of clinical features and risk type. **(C)** The nomogram based on gender and risk score for predicting 1-, 3-, and 5-year survival. **(D)** Calibration curve of 1-, 3-, and 5-year survival. **(E)** DCA of nomogram, gender, and risk score. *P < 0.05, ***P < 0.001.

Using univariate and multivariate Cox regression analyses, we evaluated the relation of clinical features and risk type with prognosis. The result displayed that only gender and risk type were independent risk factors in both univariate and multivariate analyses (**Figures 4A, B**). Gender had a hazard ratio (HR) of 2.2 in both univariate and multivariate analyses (P = 0.019 and P = 0.02, respectively). Risk type had HR of 3.7 (P = 1.1e-6) and 3.6 (P = 2.1e-6) in univariate and multivariate analyses, respectively. Therefore, we included gender and risk score to construct the nomogram (**Figure 4C**). Compared with gender, risk score contributed the more total points in the nomogram. Calibration curve analysis suggested that the predicted 1-, 3-, and 5-year survival was highly accordant with the observed survival (**Figure 4D**). In addition, decision curve analysis was implemented to evaluate the benefit that patients may obtain from gender, risk score, and nomogram. As a result, nomogram performed a more favorable performance than gender and risk score (**Figure 4E**). Consequently, the nomogram based on gender and risk score was more efficient to predict the prognosis of NPC patients.

### Construction of miRNA–mRNA competing endogenous RNA networks

**Figure 5:**
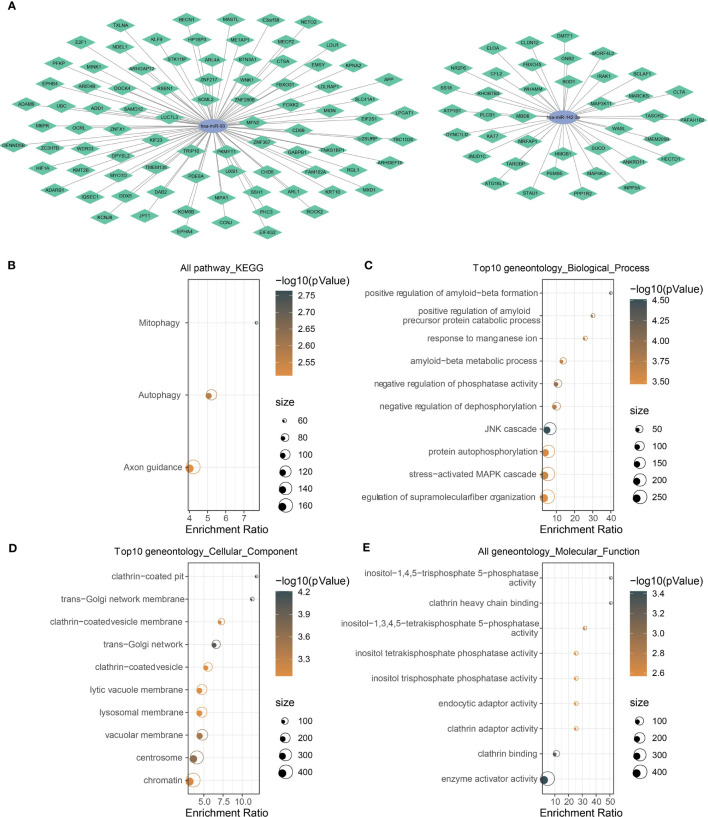
Analysis of the target genes of has-miR-142-3p and has-miR-93. **(A)** The mRNA–miRNA ceRNA networks. Green rhombus indicates target mRNAs, and ellipse indicates miRNAs. **(B)** KEGG and **(C–E)** GO functional analyses of potential target mRNAs. The color of dots indicate the significance of P values, and the dot size indicates the gene counts.

In the above sections, we identified two key miRNAs (hsa-miR-142-3p and hsa-miR-93) that had a close relation with NPC prognosis. To understand the potential molecular mechanism of the miRNAs, we applied 12 tools (starbase, PITA, TargetMiner, miRanda, microT, miRmap, miRtarbase, mircode, TargetScan, RNA22, miRDB, PicTar) to predict the potential targets of two miRNAs. The results output 39 target genes of hsa-miR-142-3p and 86 target genes of hsa-miR-93, which were visualized in ceRNA networks (**Figure 5A**). KEGG analysis demonstrated the involvement of these target genes in mitophagy and autophagy (**Figure 5B**). The top 10 enriched terms of cellular component and molecular function, as well as biological process, were visualized using GO function analysis (**Figures 5C–E**). For example, biological process terms of positive regulation of amyloid−beta metabolic process, amyloid−beta formation, and negative regulation of phosphatase activity were enriched (**Figure 5C**). Cellular component terms of clathrin-coated pit and trans-Golgi network membrane were enriched (**Figure 5D**). Molecular function terms of clathrin adaptor activity and clathrin heavy chain binding were enriched (**Figure 5E**).

### Establishing a mRNA prognostic model based on key target genes of hsa-miR-142-3p and hsa-miR-93

**Figure 6:**
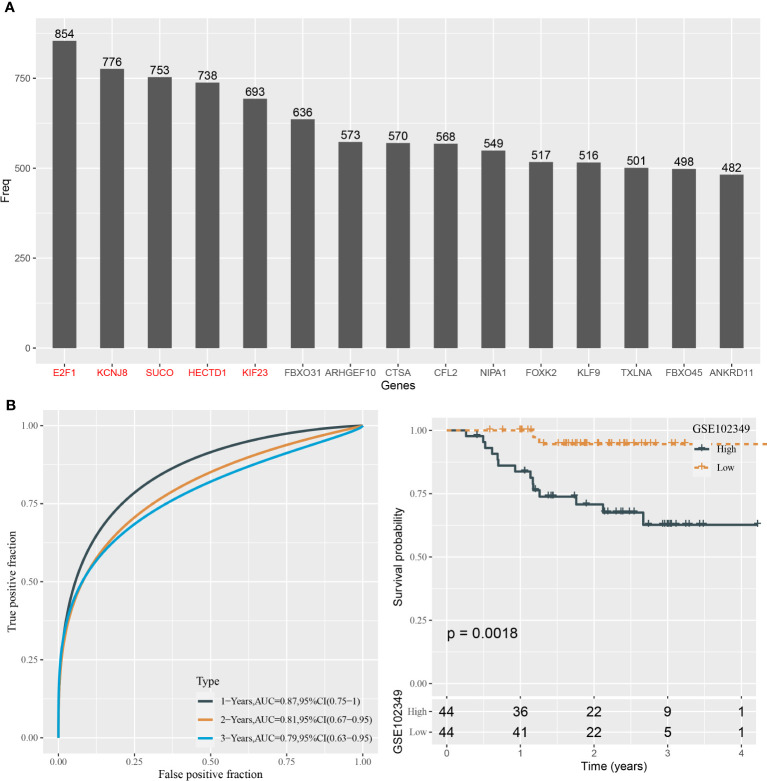
Construction of the prognostic model based on the target genes in the GSE102349 dataset. **(A)** The top 15 target genes associated with prognosis from 1,000-times random sampling. **(B)** ROC analysis and survival analysis of the five-gene prognostic model.

We predicted a total of 125 potential target genes of hsa-miR-142-3p and hsa-miR-93. Then, we used the univariate Cox regression model to analyze the relation between target genes and overall survival. Random sampling for 1,000 times from the samples of the GSE102349 dataset was performed. The target genes closely related to overall survival were remained (P < 0.05) and were ranked by the occurring frequency from 1,000-times analysis. The top five frequent target genes were selected as key target genes for establishing the mRNA prognostic model (**Figure 6A**). The coefficients of five target genes were calculated by multivariate analysis. Finally, the prognostic model was defined as the following: mRNA risk score = 1.333* E2F1 - 1.766*KCNJ8 + 1.075*SUCO - 1.030* HECTD1 - 0.340*KIF23.

The risk score for each sample in the GSE102349 dataset was determined with the mRNA prognostic model. ROC curve analysis showed that the model was efficient in predicting 1-, 3-, and 5-year survival, with AUCs of 0.87, 0.81, and 0.79, respectively (**Figure 6B**). The median risk score value was used in classifying NPC samples into two groups, high-risk and low-risk groups. Kaplan–Meier survival curves of two risk groups showed that they had an apparently different prognosis (P = 0.0018, **Figure 6B**). Hence, the five target genes could be validated by the above results to be closely involved in the prognosis.

### Immune characteristics of high- and low-risk groups

**Figure 7:**
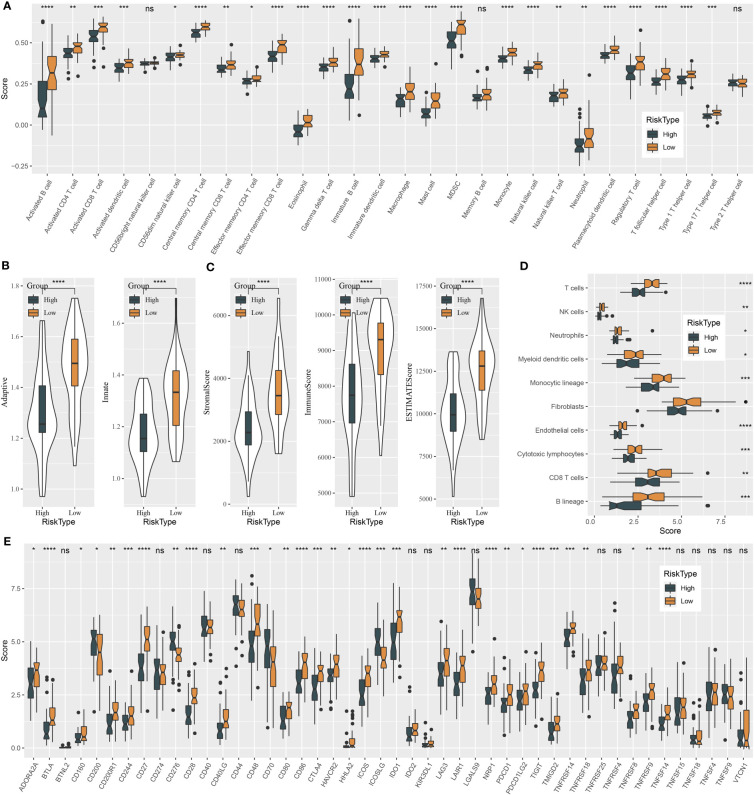
Immune characteristics of high-risk and low-risk groups in the GSE102349 dataset. **(A)** The ssGSEA score of 22 immune-related cells in two risk groups. **(B)** The ssGSEA score of adaptive and innate immune response in two risk groups. **(C)** ESTIMATE analysis of immune infiltration and stromal infiltration. **(D)** MCP-counter analysis for estimating the enrichment of 10 immune-related cells. **(E)** Expressions of immune checkpoint genes in two risk groups. ns, not significant. *P < 0.05, **P < 0.01, ***P < 0.001, ****P < 0.0001.

The tumor microenvironment plays a central role in antitumor response and immunotherapeutic response. We used several tools to assess the immune cell component, as well as immune and stromal infiltration, and analyzed immune checkpoint genes for their expression levels. Two risk groups showed a significantly different immune microenvironment. We estimated an ssGSEA enrichment score of 28 immune-related cells and found that 25 of them had a differential enrichment score, such as myeloid-derived suppressor cells (MDSCs), activated CD8 T cells, regulatory T cells, natural killer cells, activated B cells, and macrophages (**Figure 7A**). Most immune cells showed a higher abundance in the group with a low risk. In addition, ssGSEA also revealed higher enrichment of both adaptive and innate immune response scores in the low-risk group (P < 0.0001, **Figure 7B**). The ESTIMATE algorithm was used to evaluate stromal and immune infiltration of two groups, and not surprisingly, the low-risk group showed both higher immune score and stromal score than the high-risk group (**Figure 7C**, P < 0.0001). Moreover, MCP-counter was employed to dig out similar results with ssGSEA. A total of 10 types of immune-related cells all showed a higher enrichment score in the low-risk group (**Figure 7D**). Immune checkpoint expression levels also had an extreme difference in two risk groups that most of immune checkpoints, such as CTLA4, TIGIT, LAG3, and PCDC1, were more highly expressed in the low-risk group than in the high-risk group (**Figure 7E**). Immune checkpoints’ differential expression may contribute to different antitumor immune responses. The distinct immune microenvironment suggested that the five prognostic genes may play critical roles in immune modulation.

### Analysis of biological pathways, immunotherapeutic response, and drug sensitivity in two risk groups

**Figure 8:**
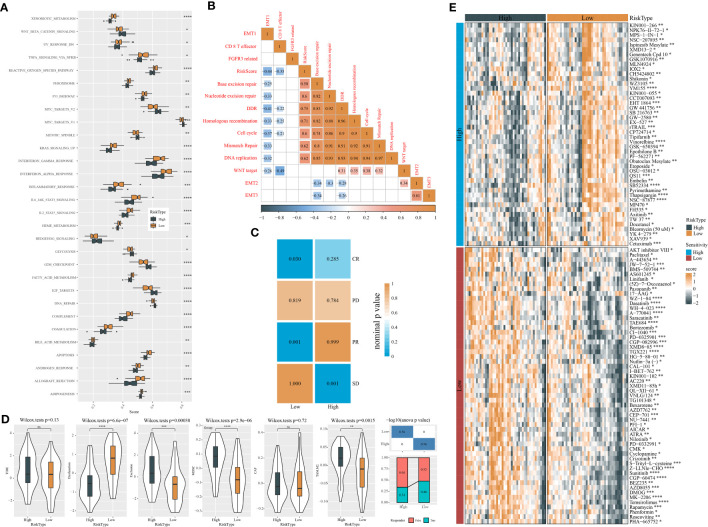
Analysis of biological pathways, response to immunotherapy, and drug sensitivity of two risk groups in the GSE102349 dataset. **(A)** Screening differentially enriched pathways between two risk groups based on ssGSEA. **(B)** Correlation analysis of risk score with 13 tumor-related pathways. Orange and blue indicate positive and negative correlations, respectively. **(C)** SubMap analysis of expression data of GSE102349 and IMvigor210 (anti-PD-L1 treatment) datasets. **(D)** TIDE analysis of two risk groups for assessing immune escape and response to immunotherapy. **(E)** The sensitivity to chemotherapeutic drugs predicted by the pRRophetic package. *P < 0.05, **P < 0.01, ***P < 0.001, ****P<0.0001.

To further understand the molecular mechanisms contributing different prognoses of two risk groups, the enrichment score of pathways from the “h.all.v7.4.symbols.gmt” file was calculated using ssGSEA. There were 30 pathways differentially activated in the two risk groups (**Figure 8A**). Immune-related pathways, for instance, interferon alpha response, interferon gamma response, inflammation response, IL2-STAT5 signaling, IL6-JAK-STAT3 signaling, and complement, were significantly more activated in the low-risk group, which was consistent with the result of immune analysis. Cell cycle-related pathways such as E2F targets, MYC target V2, MYC target V1, and G2M checkpoint were less enriched in the low-risk group. In addition, some oncogenic pathways were more activated in the high-risk group, such as Wnt signaling and Hedgehog signaling. Notably, apoptosis was more enriched in the low-risk group, which was associated with good prognosis. Risk score also manifested significant correlations with the above pathways (**Figure S2**).

Gene sets of 13 tumor-related pathways were obtained from a previous study, and their enrichment scores were calculated using ssGSEA. Pearson correlation analysis uncovered a negative association of risk score with DNA repair-related pathways including DDR (R = -0.41), base excision repair (R = -0.25), nucleotide excision repair (R = -0.33), homologous recombination (R = -0.33), and mismatch repair (R = -0.33) (**Figure 8B**).

Next, we evaluated the response of two risk groups to chemotherapy and immunotherapy. The similarity of expression profiles between GSE102349 and IMvigor210 (treated by PD-L1 inhibitors) datasets was shown by SubMap analysis. Higher similarity between two datasets indicates higher sensitivity to PD-L1 inhibitors. The results presented that the low-risk group had a higher similarity with CR and PR groups (P < 0.05, **Figure 8C**), implying that the low-risk group could obtain more benefit from immunotherapy. Furthermore, the TIDE algorithm was implemented to predict immune escape to immune checkpoint inhibitors. Two risk groups showed no significant difference of TIDE score (**Figure 8D**). However, the low-risk group exhibited more severe T-cell dysfunction than the high-risk group in which enrichment of MDSCs and M2 tumor-associated macrophages (TAM) was higher, contributing to its higher T-cell exclusion. In the response to chemotherapeutic drugs, we screened a total of 103 drugs including 46 drugs such as YM155 and vinorelbine sensitive to high-risk groups and 57 drugs such as sunitinib and temsirolimus sensitive to the low-risk group (**Figure 8E**).

### The analysis of endocrine metabolism

**Figure 9:**
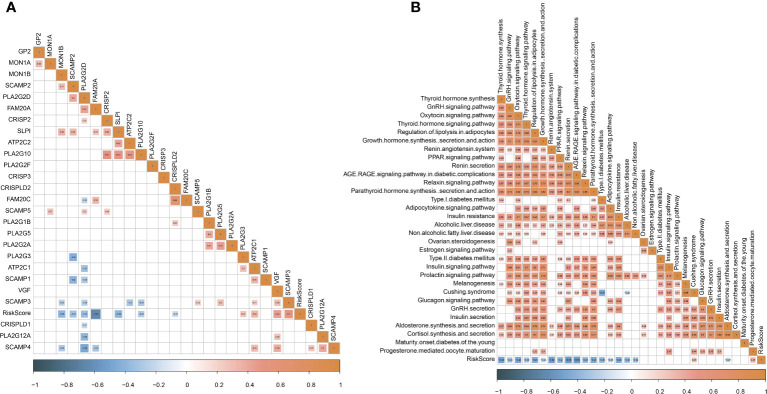
Analysis of endocrine metabolism. **(A)** Pearson analysis between endocrine-related genes and RiskScore. **(B)** Pearson analysis between endocrine pathways and RiskScore.

Abnormal endocrine metabolism is one of the complications of tumor treatment. As shown in **Figure 9A**, endocrine-related genes, such as MON1B, SCAMP2, and FAM20A, were negatively associated with RiskScore; SCAMP3 was positively correlated with RiskScore. Pearson analysis between endocrine pathways and RiskScore showed that most endocrine pathways were negatively related to RiskScore (**Figure 9B**).

### The performance of the mRNA prognostic model in immunotherapy datasets

**Figure 10:**
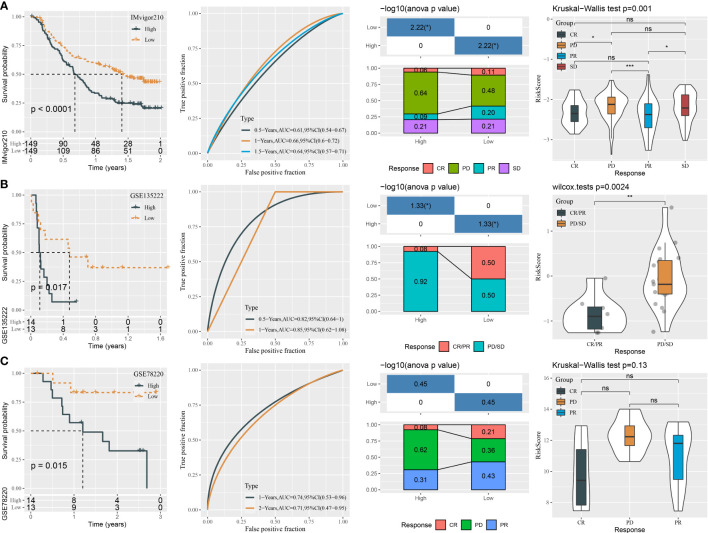
The performance of the mRNA prognostic model in three independent immunotherapy datasets. **(A–C)** ROC analysis, survival analysis, the distribution responder groups in two risk groups, and the risk score of responder groups in the IMvigor210 **(A)**, GSE135222 **(B)**, and GSE78220 **(C)** datasets. ns P>0.05, *P < 0.05, **P < 0.01, ***P < 0.001.

To further examine the exhibition of the five-gene prognostic model, we introduced three independent datasets containing patients administrated by immunotherapy (IMvigor210, GSE135222, and GSE78220). Using the same calculation method, we measured the risk score of each patient in three datasets. In the IMvigor210 dataset, the high-risk group displayed apparently worse prognosis than the low-risk group (P < 0.0001, **Figure 10A**). The prediction efficiency was evaluated by ROC, with AUCs of 0.61, 0.66, and 0.64 at 0.5-, 1-, and 1.5-year survival, respectively (**Figure 10A**). In addition, we analyzed the proportion of different response groups in two risk groups. The low-risk group had a higher proportion of CR and PR groups (11% and 20%, respectively), compared with the high-risk group (6% and 9%, respectively). The CR and PR groups also showed a lower risk score than PD and SD groups. In the GSE135222 and GSE78220 datasets, we observed correspondent results (**Figures 10B, C**). The GSE135222 dataset showed AUCs of 0.82 and 0.85 at 0.5- and 1-year survival, respectively. The low-risk group exhibited a markedly higher percentage of CR/PR patients compared with the high-risk group (50% versus 8% in low- versus high-risk groups). The GSE78220 dataset showed AUCs of 0.74 and 0.71 at 1- and 2-year survival, respectively. Expectedly, the CR and PR groups were more accumulated in the low-risk group (21% and 43%) compared with the high-risk group (8% and 31%). Moreover, the CR/PR group showed a lower risk score than the PD/SD group in both GSE135222 and GSE78220 datasets, but the difference was not significant in the GSE78220 dataset. The above observation suggested that patients with a low risk score were more sensitive to immunotherapy and could attain longer survival.

## Discussion

MiRNAs are considered as potential therapeutic targets for NPC treatment, and till now abundant miRNAs have been unveiled to be dysregulated in NPC patients (19). MiRNAs serve as importantly regulatory roles in gene expression and pathway modulation. In this study, we dug out the potential key miRNAs and mRNAs that were probably responsible for NPC development and progression. We interpreted the association between key miRNAs and mRNAs and decoded the relationships of prognostic miRNA-related mRNAs with tumor microenvironment, immunotherapy, and functional pathways.

To begin with, we deciphered the miRNA expression data and screened 332 aberrantly expressed miRNAs in NPC samples compared with normal samples. By using WGCNA, we further identified two key miRNAs (hsa-miR-142-3p and hsa-miR-93) strongly related to NPC prognosis and phenotype. We constructed a miRNA risk model based on hsa-miR-142-3p and hsa-miR-93. The risk model exhibited an intensive relation with NPC prognosis in two independent datasets (GSE32960 and GSE70970). Patients with a high risk showed significantly worse OS and DFS than the low-risk group. In addition, the risk model was also effective to predict OS in NPC samples with different clinical characteristics including ages, T stage, N1–N3 stage, and AJCC stage I–IV. These results demonstrated that hsa-miR-142-3p and hsa-miR-93 were highly responsible for NPC development.

Moreover, RiskScore was negatively correlated with endocrine genes, especially FAM20A, which had been important for endocrine-related tumors (53). The FAM20 family of kinases is a newly discovered class of secreted kinases that are capable of phosphorylating secreted proteins and proteoglycans (54). FAM20A may play a more complex role in gliomas, as correlations between FAM20A genes and low-grade gliomas have been found (55). Combining the known literature and the results of this study, it is suggested that endocrine gene FAM20A may be closely related to NPC.

The two key miRNAs have been reported in the contribution of other cancer types. For example, in esophageal squamous cell carcinoma (ESCA), hsa-miR-142-3p was identified as a prognostic biomarker (56). Non-small cell lung cancer (NSCLC) cells could be promoted by overexpression of miR-142-3p *via* interfering TGFβR1 expression (57). However, Dong et al. revealed that miR-142-3p suppressed the growth of human cervical cancer cells by attenuating HMGB1 expression levels (58). Moreover, Sharma et al. excavated that miR-142-3p functioned as a tumor-suppressive miRNA through modulating the expression of HMGA1, A2, B1, and B3 in human cervical cancer (59). miR-142-3p in different cancer types showed a discord in expression that miR-142-3p was suggested to play complicated roles (both promotive and suppressive) by interacting with specific pathways and genes in different cancers. In our study, the miR-142-3p level was significantly decreased in NPC samples, implying that high-expressed miR-142-3p may facilitate the progression of NPC.

The role of hsa-miR-93 has also been uncovered in different cancer types. For instance, a miRNA microarray result showed that miR-93 was downregulated in human colon cancer stem cells and overexpressing miR-93 strikingly inhibited cell proliferation and colony formation (60). In triple-negative breast cancer cells, cell migratory capability and invasive potential could be weakened by overexpression of mature miR-93-5p possibly by targeting WNK1 (61). In uterine cancer, the high miRNA-93 expression group had an evidently higher survival rate than the low miRNA-93 expression group (62). The results of the above studies were accordant with our study that miRNA-93 expression was elevated in the NPC group compared with the normal group.

To clarify the potential mechanisms of the two miRNAs in NPC, their potential target genes (mRNAs) were predicted by utilizing 12 different tools to build ceRNA networks. As a result, we confirmed 39 target genes of miR-142-3p and 86 target genes of miR-93. KEGG analysis revealed that these target genes were significantly involved in autophagy and mitophagy. Autophagy occurs under stressful situations such as the presence of abnormal proteins and nutrient deprivation, which degrades cellular proteins and organelles to provide precursors for recycling (63). Some clinical trials have presented that inhibiting autophagy has feasible benefits in multiple cancer types such as glioblastoma, melanoma, and pancreatic cancer (64). Autophagy and mitophagy are demonstrated to contribute to the reprogramming of cancer metabolism that is a major challenge for anticancer therapy (65). Therefore, we supposed that maybe one of the mechanisms of miR-142-3p and miR-93 in NPC development was their participation in autophagy and mitophagy process responsible for cancer metabolism.

In order to distinguish key target genes of the two miRNAs, we applied random sampling and univariate Cox regression on 125 potential target genes. As a consequence, we confirmed five target genes that had prognostic effects on NPC, namely, E2F1, KCNJ8, SUCO, HECTD1, and KIF23, where SUCO and HECTD1 are the targets of miR-142-3p and E2F1, KCNJ8, and KIF23 are the targets of miR-93. E2F1 has been widely reported to regulate cell cycle and cell death and has a significant role in multiple cancer types. E2F1 target pathways are considered as important targets for cancer treatment (66). Limited studies of cancer have been found related to the other four genes. Based on these five target genes, we further established a prognostic model. The five-gene prognostic model manifested substantial performance in predicting 1-, 2-, and 3-year survival with AUCs of 0.87, 0.81, and 0.79 respectively. According to the model, high-risk and low-risk groups were defined with disparate prognosis.

We further investigated the differences of the tumor microenvironment and functional pathways between two risk groups for excavating the biological influence of the five target genes in NPC. Two risk groups had distinct immune cell infiltration. Anticancer immune cells, for instance, activated B cells, NK cells, dendritic cells, and CD8 T cells, were evidently higher in the low-risk group compared with the high-risk group, which led to the stronger anticancer response and clearance of cancer cells. The high-risk group showed both higher innate and adaptive immune response than the low-risk group. Analysis of biological pathways unveiled that the low-risk group displayed higher activation of immune-related pathways, for instance, IL2-STAT5 signaling, interferon alpha response, interferon gamma response, IL6-JAK-STAT3 signaling, inflammation response, and complement. Importantly, DNA repair pathways, cell cycle-related pathways, and apoptosis were also more enriched in the low-risk group, supporting that the two key miRNAs may have an interaction with these target genes. However, the specific association between the miRNAs and five target genes should be further validated in future experiments.

The different proportion of tumor-infiltrating immune cells has a profound effect on both cancer prognosis and immunotherapy (67). SubMap analysis predicted that the low-risk group was more sensitive to ICB therapy than the high-risk group, which may result from the higher expression of critical immune checkpoints such as CTLA4, IDO1, LAG3, and PDCD1 (PD-1) in the low-risk group. Anti-PD-1/PD-L1 therapy has shown some favorable outcomes in clinical trials of NPC (68). Our five-gene model can help clinicians to better select the patients sensitive to ICB therapy and raise the efficiency of immunotherapy.

## Conclusions

In conclusion, this study identified two key miRNAs (miR-142-3p and miR-93) and predicted their potential key target genes. MiR-142-3p and miR-93 contributed to NPC survival possibly through regulating autophagy pathways. In addition, we confirmed five prognostic target genes (E2F1, KCNJ8, SUCO, HECTD1, and KIF23) and constructed a five-gene prognostic model. The model was effective to predicting NPC prognosis and could provide a guidance for personalized immunotherapy in NPC patients.

## Data availability statement

The datasets presented in this study can be found in online repositories. The names of the repository/repositories and accession number(s) can be found in the article/**Supplementary Material**.

## Author contributions

All authors contributed to this present work. XXZ, XiaL and CW designed the study, SW acquired the data. YZ and BL drafted the manuscript. XinL revised the manuscript. All authors contributed to the article and approved the submitted version.
