# Supplementary material for: Identification of markers for predicting prognosis and endocrine metabolism in nasopharyngeal carcinoma by miRNA–mRNA network mining and machine learning
Source: Front Endocrinol (Lausanne). 2023 Jul 19;14:1174911. doi: 10.3389/fendo.2023.1174911 (PMC10396331; doi:10.3389/fendo.2023.1174911)
Supplement: Supplementary file 1 [file DataSheet_1.zip › Supplementary captions.DOCX]

Supplementary materials

Figure S1. A: Multivariate analysis of has-miR-142-3p and has-miR-93 in GSE32960 dataset. B: the mRNA expression of has-miR-142-3p in NPC cell lines. C: the mRNA expression of has-miR-93 in NPC cell lines.

Figure S2. Pearson correlation analysis between risk score and biological pathways. Orange and grey indicate positive and negative correlations respectively. × indicates no statistical difference.

Table S1. The list of 332 DEmiRNAs

Table S2. The clinical information and comparison of training and test groups in GSE32960 dataset
