# Supplementary material for: Identification of markers for predicting prognosis and endocrine metabolism in nasopharyngeal carcinoma by miRNA–mRNA network mining and machine learning
Source: Front Endocrinol (Lausanne). 2023 Jul 19;14:1174911. doi: 10.3389/fendo.2023.1174911 (PMC10396331; doi:10.3389/fendo.2023.1174911)
Supplement: Supplementary file 1 [file DataSheet_1.zip › Table S1.DOCX]

Table S1. The clinical information and comparison of training and test groups in GSE32960 dataset

|  | Test (N=125) | Train (N=187) | P-value |
| --- | --- | --- | --- |
| OS |  |  |  |
| 0 | 94 (75.2%) | 144 (77.0%) | 0.817 |
| 1 | 31 (24.8%) | 43 (23.0%) |  |
| T.stage |  |  |  |
| T1 | 19 (15.2%) | 47 (25.1%) | 0.096 |
| T2 | 41 (32.8%) | 48 (25.7%) |  |
| T3 | 33 (26.4%) | 38 (20.3%) |  |
| T4 | 32 (25.6%) | 54 (28.9%) |  |
| N.stage |  |  |  |
| N0 | 18 (14.4%) | 26 (13.9%) | 0.913 |
| N1 | 61 (48.8%) | 87 (46.5%) |  |
| N2 | 29 (23.2%) | 43 (23.0%) |  |
| N3 | 17 (13.6%) | 31 (16.6%) |  |
| Stage |  |  |  |
| I | 4 (3.2%) | 8 (4.3%) | 0.694 |
| II | 33 (26.4%) | 53 (28.3%) |  |
| III | 41 (32.8%) | 50 (26.7%) |  |
| IV | 47 (37.6%) | 76 (40.6%) |  |
| Age |  |  |  |
| <=45 | 54 (43.2%) | 94 (50.3%) | 0.267 |
| >45 | 71 (56.8%) | 93 (49.7%) |  |
| Gender |  |  |  |
| Female | 36 (28.8%) | 43 (23.0%) | 0.306 |
| Male | 89 (71.2%) | 144 (77.0%) |  |
