# Supplementary figures and images for: Identification of markers for predicting prognosis and endocrine metabolism in nasopharyngeal carcinoma by miRNA–mRNA network mining and machine learning
Source: Front Endocrinol (Lausanne). 2023 Jul 19;14:1174911. doi: 10.3389/fendo.2023.1174911 (PMC10396331; doi:10.3389/fendo.2023.1174911)

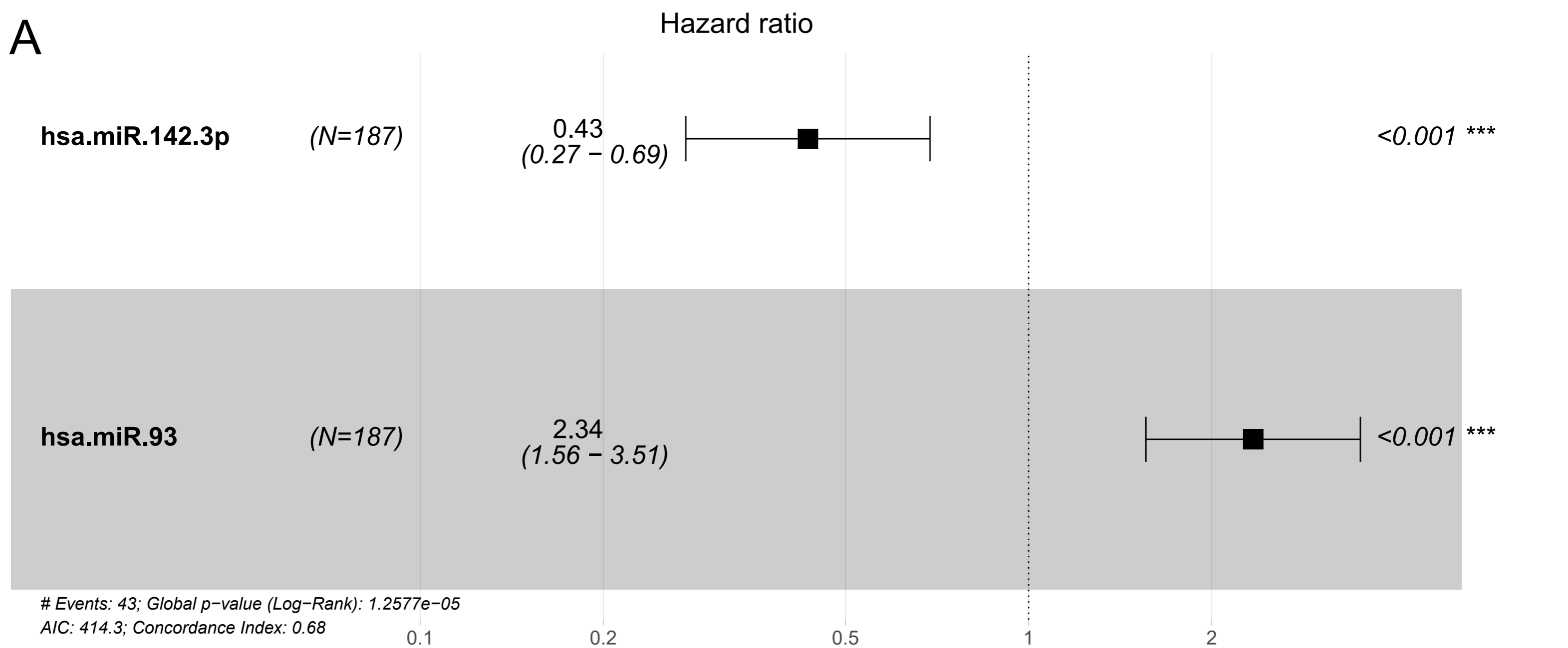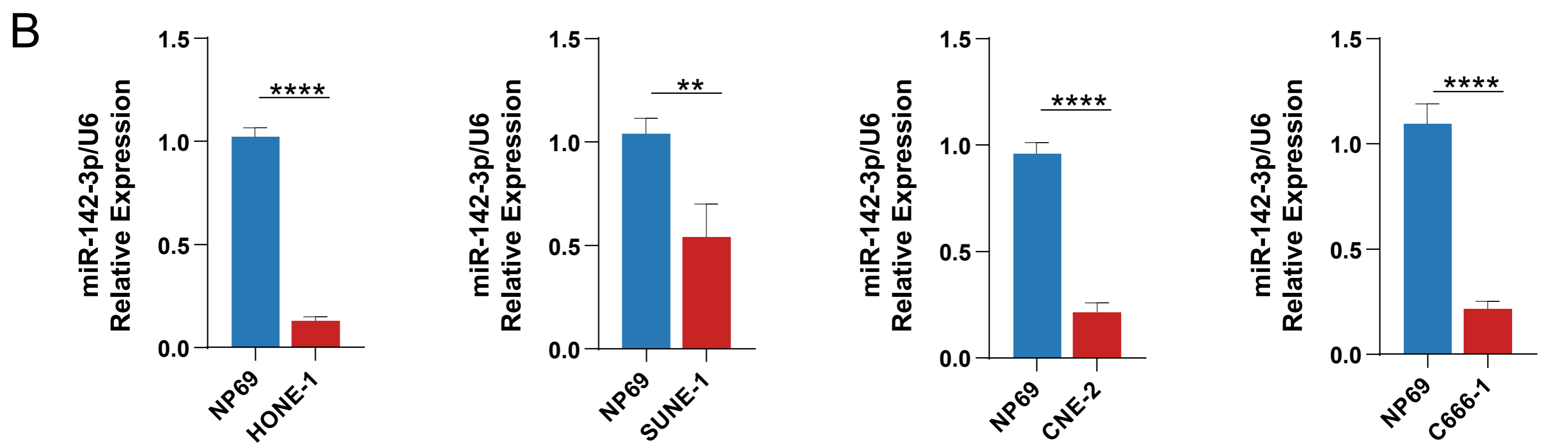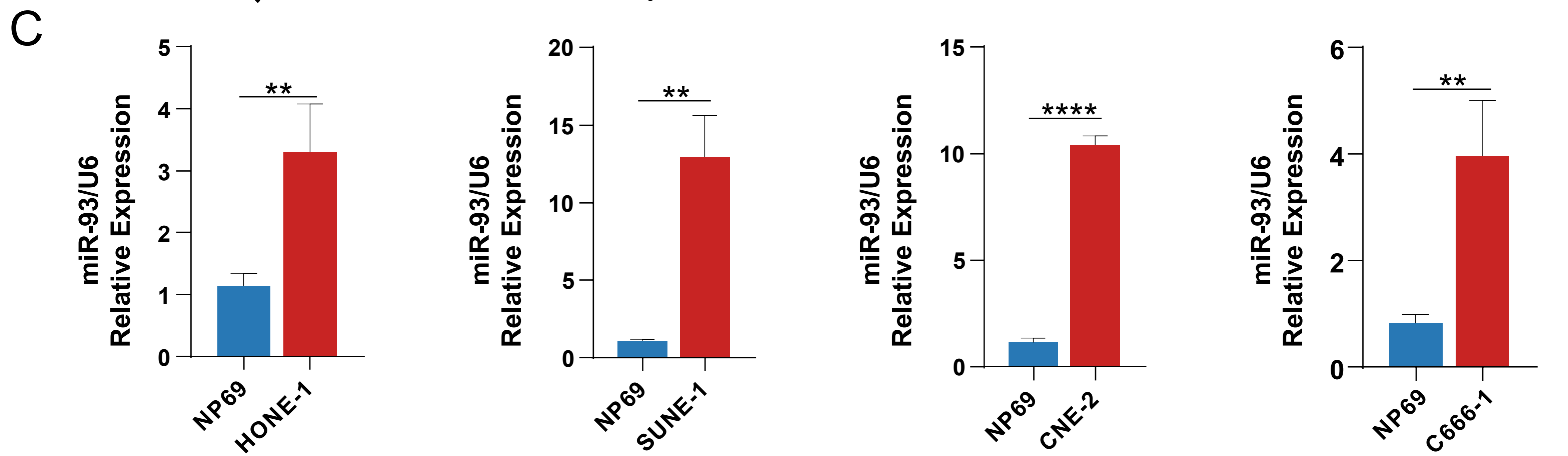

Supplement: Supplementary file 1 [file DataSheet_1.zip › Supplementary Figure 1.PDF]

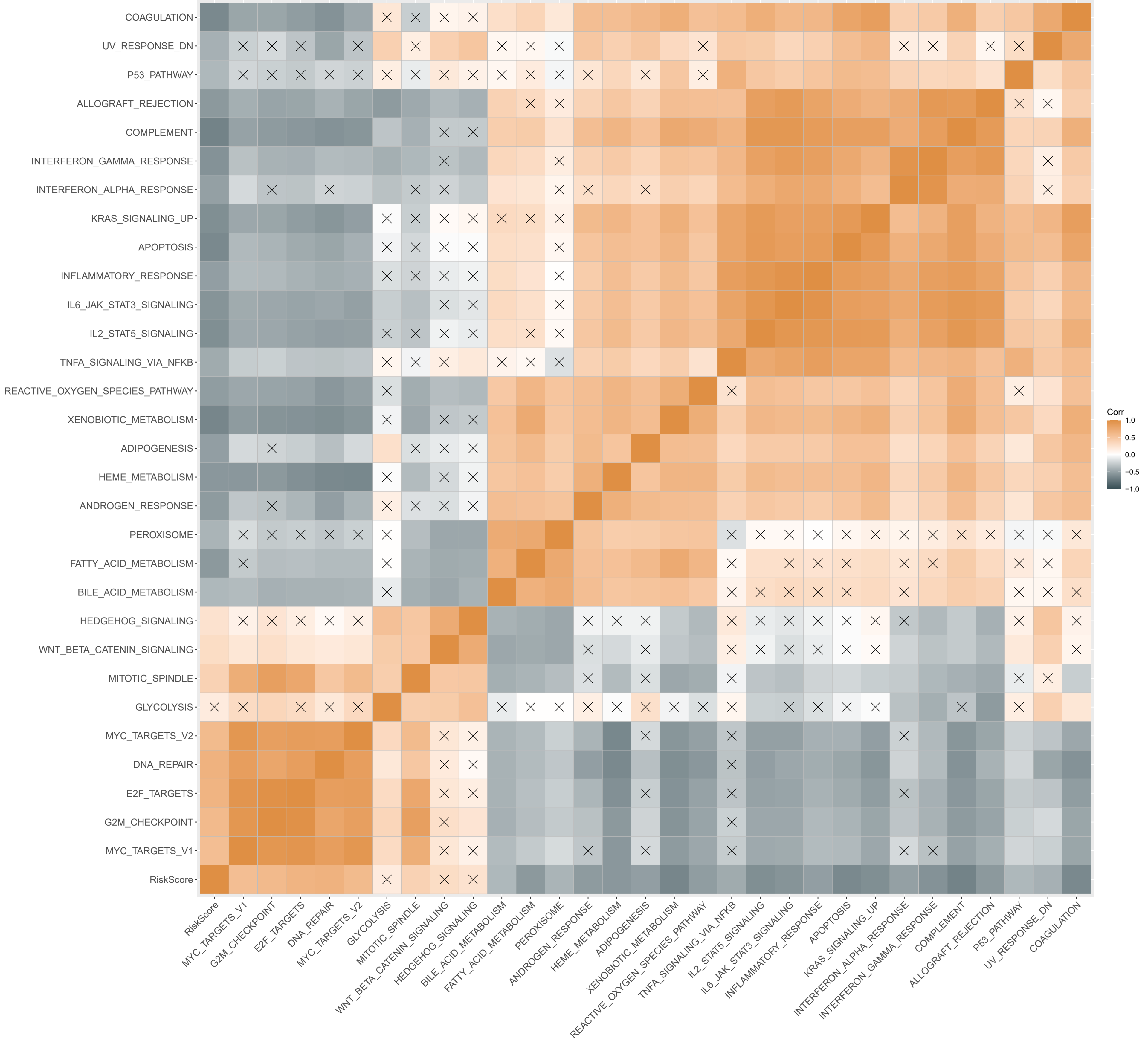

Supplement: Supplementary file 1 [file DataSheet_1.zip › Supplementary Figure 2.PDF]
